# Active DNA Demethylase, TET1, Increases Oxidative Phosphorylation and Sensitizes Ovarian Cancer Stem Cells to Mitochondrial Complex I Inhibitor

**DOI:** 10.3390/antiox13060735

**Published:** 2024-06-17

**Authors:** Lin-Yu Chen, Yao-An Shen, Ling-Hui Chu, Po-Hsuan Su, Hui-Chen Wang, Yu-Chun Weng, Shiou-Fu Lin, Kuo-Chang Wen, Phui-Ly Liew, Hung-Cheng Lai

**Affiliations:** 1 Department of Obstetrics and Gynecology, Shuang Ho Hospital, Taipei Medical University, New Taipei City 23561, Taiwan; 20016@s.tmu.edu.tw (L.-Y.C.); b89401059@ntu.edu.tw (L.-H.C.); 19345@s.tmu.edu.tw (K.-C.W.); 2 Department of Pathology, School of Medicine, College of Medicine, Taipei Medical University, Taipei 11031, Taiwan; shen1202@tmu.edu.tw; 3 Graduate Institute of Clinical Medicine, School of Medicine, College of Medicine, Taipei Medical University, Taipei 11031, Taiwan; 4 College of Health Technology, National Taipei University of Nursing and Health Sciences, Taipei 11219, Taiwan; pohsuan@ntunhs.edu.tw; 5 Department of Obstetrics and Gynecology, School of Medicine, College of Medicine, Taipei Medical University, Taipei 11031, Taiwan; 6 Department of Obstetrics and Gynecology, Tri-Service General Hospital, National Defense Medical Center, Taipei 11490, Taiwan; 7 Translational Epigenetics Center, Shuang Ho Hospital, Taipei Medical University, New Taipei City 23561, Taiwan; 8 Department of Pathology, Shuang Ho Hospital, Taipei Medical University, New Taipei City 23561, Taiwan; 18011@s.tmu.edu.tw

**Keywords:** ten-eleven translocation 1 (TET1), DNA demethylation, ovarian cancer stem cells, oxidative phosphorylation (OXPHOS), mitochondria, mitochondrial complex I inhibitor, casein kinase-2 subunit alpha (CK2α) inhibitor

## Abstract

Ten-eleven translocation 1 (TET1) is a methylcytosine dioxygenase involved in active DNA demethylation. In our previous study, we demonstrated that TET1 reprogrammed the ovarian cancer epigenome, increased stem properties, and activated various regulatory networks, including metabolic networks. However, the role of TET1 in cancer metabolism remains poorly understood. Herein, we uncovered a demethylated metabolic gene network, especially oxidative phosphorylation (OXPHOS). Contrary to the concept of the Warburg effect in cancer cells, TET1 increased energy production mainly using OXPHOS rather than using glycolysis. Notably, TET1 increased the mitochondrial mass and DNA copy number. TET1 also activated mitochondrial biogenesis genes and adenosine triphosphate production. However, the reactive oxygen species levels were surprisingly decreased. In addition, TET1 increased the basal and maximal respiratory capacities. In an analysis of tricarboxylic acid cycle metabolites, TET1 increased the levels of α-ketoglutarate, which is a coenzyme of TET1 dioxygenase and may provide a positive feedback loop to modify the epigenomic landscape. TET1 also increased the mitochondrial complex I activity. Moreover, the mitochondrial complex I inhibitor, which had synergistic effects with the casein kinase 2 inhibitor, affected ovarian cancer growth. Altogether, TET1-reprogrammed ovarian cancer stem cells shifted the energy source to OXPHOS, which suggested that metabolic intervention might be a novel strategy for ovarian cancer treatment.

## 1. Introduction

Ovarian carcinoma is the most lethal gynecological malignancy because of the nonspecific symptoms in the early disease stages and delayed diagnoses until the advanced stages. Surgery followed by adjuvant chemotherapy remains the mainstay of treatment. Variation in individual response is common due to tumor heterogeneity; however, most patients with ovarian cancer develop recurrent and chemoresistant tumors. Therefore, understanding the biology of chemoresistance is important to improve the survival of patients with ovarian cancer. New and more precise therapeutic strategies are needed. Although The Cancer Genome Atlas (TCGA) consortium has revealed the genomic changes in ovarian cancer, no driver mutations were identified that could lead to the development of target therapeutics thus far.

In addition to genetics, epigenetics has been proven as a driving force in cancer development. DNA methylation, including global hypomethylation and gene-specific hypermethylation, is an important epigenetic modification that has a penetrating effect on genome stability, transcription, and development. Imbalances in genomic methylation homeostasis also contribute to cancer development and progression. More recently, findings regarding active DNA demethylation have raised new hope for cancer research. The ten-eleven translocation (TET) protein family is a family of alpha-ketoglutarate (α-KG) and Fe^2+^-dependent dioxygenases that mediate DNA demethylation [1]. This protein family has three 5-methylcytosine (5-mC) dioxygenase members, namely TET1, TET2, and TET3, that convert 5-mC to 5-hydroxymethylcytosine (5-hmC), which further triggers base excision repair and activation-induced cytidine deamination [2,3,4]. TET1 was first identified as a fusion protein [5] involved in a prerequisite step in the initiation of demethylation [1]. TET1 also binds primarily to the transcription start site of the CpG-rich promoters in the embryonic stem cell genome and regulates the level of DNA methylation [6]. Although the role of TET1 in cancer biology remains poorly understood thus far, the enzymatic role of TET proteins in the mammalian DNA demethylation pathway suggests the involvement of epigenetic dysregulation in various human cancers [7,8]. Recent investigations have suggested an oncogenic role of TET1 in solid cancers [9,10,11] and its involvement in the maintenance of cancer stem cells (CSCs) in prostate cancer [12], triple-negative breast cancer (TNBC) [13,14], and endometrial cancer [15]. In TNBC CSCs, TET1 is essential for their maintenance. It regulates the expression of OCT4 and NANOG, which are critical for the self-renewal and expansion of TNBC CSCs. TET1 also controls the cell cycle by upregulating the CDK1 and CCNB1 levels. In endometrial CSCs, TET1 increases the levels of 5-hmC in the promoter regions of the key CSC marker genes (NANOG, SOX2, and OCT4), thereby enhancing their expression and supporting the cancer stemness. However, the understanding of TET1 in ovarian cancer remains limited and controversial.

The ectopic expression of TET1 has been shown to inhibit colony formation, cell migration, and invasion [16,17]. TET1 overexpression reverses the epithelial–mesenchymal transition (EMT) process by inhibiting Wnt/β-catenin signaling through the demethylation of *SFRP2* and *DKK1*, the upstream antagonist of this pathway [18]. Conversely, higher TET1 expression in ovarian cancer is associated with poorer survival [19,20]. TET1 is significantly upregulated in cisplatin-resistant cells. The ectopic expression of TET1 promotes cisplatin resistance and reduces the cytotoxicity induced by cisplatin [20]. Additionally, TET1 re-expresses Vimentin through active DNA demethylation, contributing to a partial EMT [21]. These studies reported the phenotypic changes and single signaling pathway associated with TET1; however, the mechanisms and the full extent of its impact on ovarian cancer cells remain unclear. Our previous investigations revealed that TET1 reprogrammed the epigenomics of ovarian cancer cells, leading to a stem-like state that activated various regulatory networks, including the developmental, immune response, hormone response, and metabolic networks [9]. As the central hub of gene–gene interactions in immune response groups, casein kinase 2 (CK2) provides a therapeutic target to overcome the aggressiveness of ovarian cancer [9]. Cancer metabolism has been extensively studied, which reflects the importance of the metabolic supply for cancer cell survival, progression, and metastasis. Moreover, cancer metabolism has been suggested to affect immunometabolism and stem cell metabolism [22,23]; however, the metabolism in ovarian CSCs, especially in the TET1-reprogrammed stem state, has not yet been investigated. Elucidating ovarian CSC metabolism may offer a more beneficial therapeutic strategy for improving the overall survival of patients with ovarian cancer.

In normal cells, glucose is metabolized through glycolysis, the tricarboxylic acid (TCA) cycle, and oxidative phosphorylation (OXPHOS) to produce 34–38 molecules of adenosine triphosphate (ATP) per molecule of glucose. Cancer cells reportedly use the Warburg effect, which involves glycolysis rather than OXPHOS as an energy source [24,25]. CSCs, which are defined as subpopulations of cancer cells with a high capacity for self-renewal and the ability to initiate tumorigenesis, therapeutic resistance, and metastatic dissemination, exhibit the features of both normal stem cells and cancer cells [26]; however, the energy adaptations of CSCs remain unclear [27,28,29]. Recent reports have suggested that metabolic plasticity allows CSCs to switch between distinct metabolic preferences in different microenvironments [22,30,31,32].

The present study aimed to analyze the metabolism of TET1-reprogrammed ovarian CSCs and explore the possibility of a metabolic intervention for ovarian cancer treatment.

## 2. Materials and Methods

### 2.1. Cell Lines and Cell Culture Studies

All the cell lines (SKOV3, HeyC2, CP70, ES2, TOV21G, OVCAR3, and OVSAHO) were obtained from the American Type Cell Collection (Manassas, VA, USA). The TET1 overexpression and knockdown methods have been described previously [9]. For the drug study, the data were analyzed for drug combination effects using the CompuSyn software version 1.0, which employs the Chou–Talalay method [33]. The combination index and dose reduction index values, generated using a CompuSyn software simulation, were averaged for each cell line, and used to generate fraction affected–combination index plots for each constant–ratio drug combination.

### 2.2. Quantitative 5gmC PCR and mtDNA-CN

Total genomic DNA was isolated from TET1-transfected or control cells. For 5gmC, the hydroxylmethylated DNA was immunoprecipitated using Hydroxymethyl Collector^TM^ (Active Motif, Carlsbad, CA, USA) according to the manufacturer’s instructions. For mtDNA-CN, the mitochondrial genome was measured using *ATP6*, *COX3*, and *ND1*. The 18S ribosomal RNA was used as a reference for the normalization of the qPCR data. Quantitative 5gmC or mtDNA-CN PCR was performed using RT^2^ SYBR Green qPCR Mastermixes (SABiosciences, Qiagen, Valencia, CA, USA). The target regions were detected using the following primer sequences: *NDUFA13*: 5′-CCTGTCTGGGCAAAAGAGGAGTA-3′ (forward), 5′-ATGAGTGTGGCGGAAGCACTT-3′ (reverse); *NDUFAF3*: 5′-GGAGAGCAGGGGACGAAAT-3′ (forward), 5′-ATGGGCGGGTTCTGATGT-3′ (reverse); and *SCO2*: 5′-GCCGTCTCGATGAACACGT-3′ (forward), 5′-AGTGGACCAAGCACGAGAGG-3′ (reverse). The mtDNA-CN was assessed using the following primer sequences: *ATP6*: 5′-TTTATTGCCACAACTAACCTCCT-3′ (forward), 5′-TTGGGTGGTTGGTGTAAATG-3′ (reverse); *COX3*: 5′-GCAGGATTTTTCTGAGCCTTT-3′ (forward), 5′-GCCAGTGCCCTCCTAGTTG-3′ (reverse); *ND1*: 5′-CACCCAAGAACAGGGTTTGT-3′ (forward), 5′-TGGCCATGGGTATGTTGTTAA-3′ (reverse); and 18S rRNA: 5′-CTCAACACGGGAAACCTCAC-3′ (forward), 5′-CGCTCCACCAACTAAGAACG-3′ (reverse).

### 2.3. Seahorse XF Extracellular Flux Analyser

A mitochondrial respiration analysis was performed using the Seahorse XF Cell Mito Stress Test (Agilent, Santa Clara, CA, USA). Briefly, the ovarian cancer cells were sequentially treated with 1 μM oligomycin, 1 μM carbonyl cyanide-p-trifluoromethoxyphenylhydrazone, and a 0.5 μM mixture of rotenone and antimycin A according to the manufacturer’s instructions. The Seahorse XFe Wave Software (Agilent, version 2.6) was used to analyze the data. The OCRs were measured, followed by the sequential treatment every 3 min. The basal respiration, ATP-linked respiration, proton leak, maximal respiration, spare respiratory capacity, and non-mitochondrial respiration were assessed.

### 2.4. Intracellular ATP Level Measurement

A total of 2 × 10^4^ cells were seeded into an opaque white 96-well plate for 24 h at 37 °C with 5% CO_2_. The intracellular ATP levels were determined using the Luminescent ATP Detection Assay Kit (Catalogue number: ab113849, Abcam, Cambridge, UK) according to the manufacturer’s protocol. The fluorescence intensity after the TET1 expression compared with that in the control cells was used to calculate the relative intracellular ATP levels.

### 2.5. Mitochondrial Mass Examination

The cells were preincubated with 2.5 μM 10-N-nonyl acridine orange (Thermo Fisher Scientific, Waltham, MA, USA) for 10 min at 25 °C in the dark and harvested in a solution containing 5 mM KCl, 140 mM NaCl, 2 mM CaCl_2_, 1 mM MgCl_2_, 10 mM glucose, and 5 mM 4-(2-hydroxyethyl)-1-piperazineethanesulfonic acid buffer (pH 7.4). The fluorescence intensity was analyzed using a flow cytometer.

### 2.6. Intracellular ROS and Mitochondrial Superoxide Anion Level Measurement

The cells were incubated with Dulbecco’s Modified Eagle Medium containing CellROX Green (Thermo Fisher Scientific) or MitoSOX Red (Thermo Fisher Scientific) for 15 min at 37 °C. CellROX Green was used for the determination of the intracellular ROS levels, while MitoSOX Red was used to measure the mitochondrial superoxide anion levels. After staining, the cells were washed three times using a buffer (phosphate buffered saline, pH 7.2, 0.5% bovine serum albumin) and centrifuged for 5 min at 1500× *g*. Signals were collected for each sample in the flow cytometry analysis after gating the viable cells using forward and side scatter signals.

### 2.7. Mitochondrial Complex I Activity Measurement

The mitochondria were isolated using a Dounce homogenizer (Mitochondria Isolation Kit for Cultured Cells, Merck, Darmstadt, Germany), while the complex I activity was determined using a Mitochondrial Complex I Activity Assay Kit (Merck) according to the manufacturer’s protocol. In brief, the mitochondria were extracted using a mitochondrial buffer (1 mM ethylenediaminetetraacetic, 210 mM mannitol, 70 mM sucrose, and 5 mM Tris•HCl, pH 7.5), followed by centrifugation at 1200× *g* for 15 min at 4 °C. The supernatant was centrifuged at 17,000× *g* for 15 min at 4 °C to pellet the mitochondria. The mitochondrial fraction was resuspended for washing and centrifuged at 17,000× *g* for 15 min at 4 °C. For the complex I activity measurement, 4 μg mitochondria were added to the assay medium, and the baseline absorbance was read at 600 nm immediately. An NADH1 working solution was added and the decrease in the absorbance was recorded at 600 nm immediately. In parallel, the same quantity of reagents and samples but with the addition of rotenone (1 mM) was used.

### 2.8. Tricarboxylic Acid Cycle Metabolite Analysis

#### 2.8.1. Chemicals and Reagents

Twenty-three polar metabolite standards were obtained from Sigma-Aldrich (Merck). The stock solutions were prepared in methanol at 1 ppm (1 μg/mL) and stored at 20 °C. The standard mixture was mixed and diluted in series to the desired concentrations at an equal volume. Methanol, LC-MS grade, was obtained from J.T. Baker (Phillipsburg, NJ, USA). Formic acid, LC-MS grade, was obtained from Fisher Chemical (San Jose, CA, USA). Acetonitrile, LC-MS grade, was obtained from Merck.

#### 2.8.2. Cell Culture and Sample Preparation

The ovarian cancer cells were cultivated from approximately 1 × 10^7^ cells, and 0.8 mL of the cell sample was collected. The cells were separated by centrifuging at 1500× *g* for 5 min. The pellet was gathered for the subsequent procedure. Then, 1 mL of ice-cold methanol–water (7:3) solution was added to the cell pellets and vortexed for 1 min. Cell lysis was achieved by performing two consecutive freeze and thaw steps. The samples were centrifuged for 15 min at 15,000× *g*. The supernatant was filtered using a 0.22 μm filter.

#### 2.8.3. Chromatography Conditions

For the normal phase analysis, an ethylene-bridged hybrid (BEH) amide column, 1.7 μm (2.1 × 100 mm; Waters, Manchester, UK), was used. The column temperature was 40 °C. The same conditions were used in both the negative and positive modes. The flow rate was 0.3 mL/min with mobile phase A (water) and mobile phase B (acetonitrile, ACN), both containing 0.1% formic acid. The following elution gradient was used: 0 min, 100% B; 1 min, 100% B; 6 min, 5% B; 8.0 min, 5% B; 8.1 min, 100% B; and 10 min, 100% B.

#### 2.8.4. Mass Spectrometry

The analyses were performed using targeted protocols using the Xevo TQ-S triple quadrupole mass spectrometer and the quadrupole time-of-flight mass spectrometer (Waters SYNAPT G2 8K HDMS). The Waters ACQUITY UPLC system was connected to a Waters Xevo-TQS triple quadrupole mass spectrometer with an electrospray ionization probe (Waters Corporation, Milford, MA, USA). Source-dependent parameters were constantly maintained throughout the analysis, including the capillary voltage (2.8 kV), the desolvation temperature (450 °C), and the desolvation gas flow (900 L/h). Nitrogen (purity > 99.9%) and argon (purity > 99.998%) were used as the desolvation and collision gases, respectively. The multiple reaction monitoring (MRM) transition mode was used for the quantification of the metabolites in their individual MRM transitions. The dwell time was automatically calculated based on the region of the retention time window, the number of MRM transitions, and the number of data points required to form a peak. The Waters SYNAPT G2 8K HDMS quadrupole time-of-flight mass spectrometer was equipped with an electrospray ionization probe (Waters Corporation). The source-dependent parameters were constantly maintained throughout the analysis, including the capillary voltage (2.8 kV for the positive mode and 1.7 kV for the negative mode), the desolvation temperature (400 °C for the positive mode and 450 °C for the negative mode), and the desolvation gas flow (800 L/h for the positive mode and 900 L/h for the negative mode). The Mass Lynx 4.1 software was used for the target analysis data acquisition and integration.

### 2.9. Statistical Analysis

All the data are presented as mean ± SD. A statistical analysis was performed using the Student’s t-test. A difference between the groups with *p* < 0.05 was considered significant.

## 3. Results

### 3.1. TET1 Upregulated OXPHOS-Related Genes through Hydroxymethylation-Dependent DNA Demethylation in TET1-Reprogrammed Ovarian CSCs

**Figure 1:**
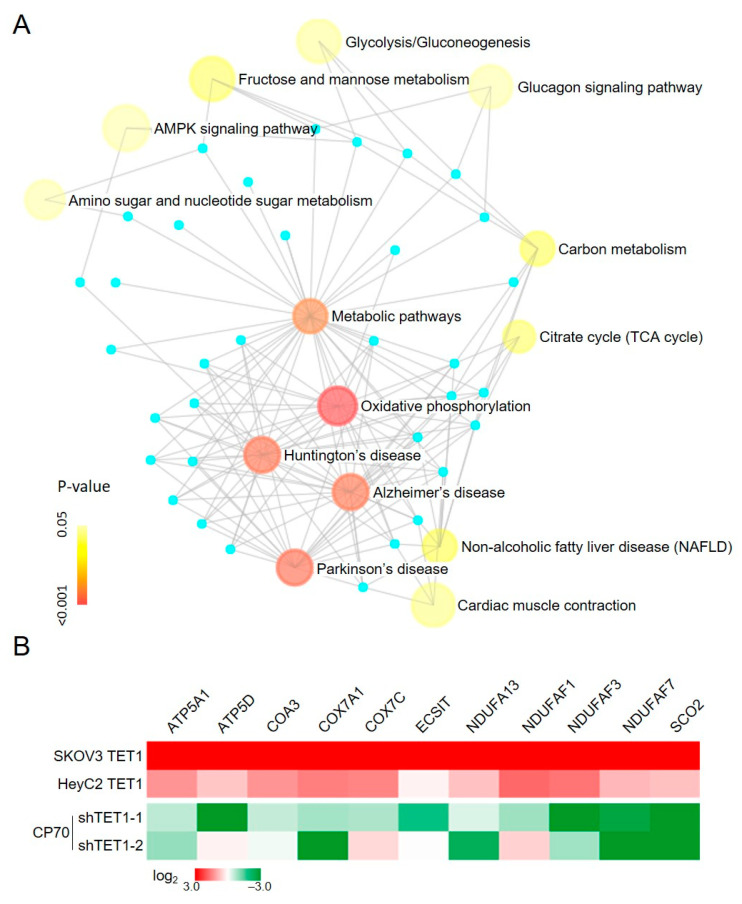
Enrichment network analysis of the TET1-mediated metabolic processes. (**A**) Enrichment map analysis, following TET1 overexpression, revealed several distinct metabolic process clusters. Nodes represent TET1-activated gene sets, edges represent mutual overlap, and light blue dots represent genes not clusters in the gene set. Node size is proportional to the total number of genes in each set, and node color represents the *p*-values. (**B**) OXPHOS-related genes were re-expressed after TET1 expression. OXPHOS-related gene expression levels were examined using quantitative reverse transcription polymerase chain reaction (qRT-PCR) in TET1-reprogrammed SKOV3 and HeyC2, as well as in TET1-knockdown CP70.

Based on our previous computational predictions [9], we analyzed the effect of TET1-reprogrammed ovarian CSCs on metabolism-related genes using the NetworkAnalyst tool [34]. The enrichment analysis showed that OXPHOS (22 hits, *p* < 0.001, FDR < 0.001) was the most important process after TET1-reprogrammed SKOV3 and HeyC2 (Figure 1A). Then, we determined the expression of the mitochondrial and nuclear-encoded OXPHOS genes using quantitative reverse transcription polymerase chain reaction (qRT-PCR). The OXPHOS-related genes were upregulated in TET1-reprogrammed SKOV3 and HeyC2 and suppressed by TET1 knockdown in CP70 (Figure 1B).

**Figure 2:**
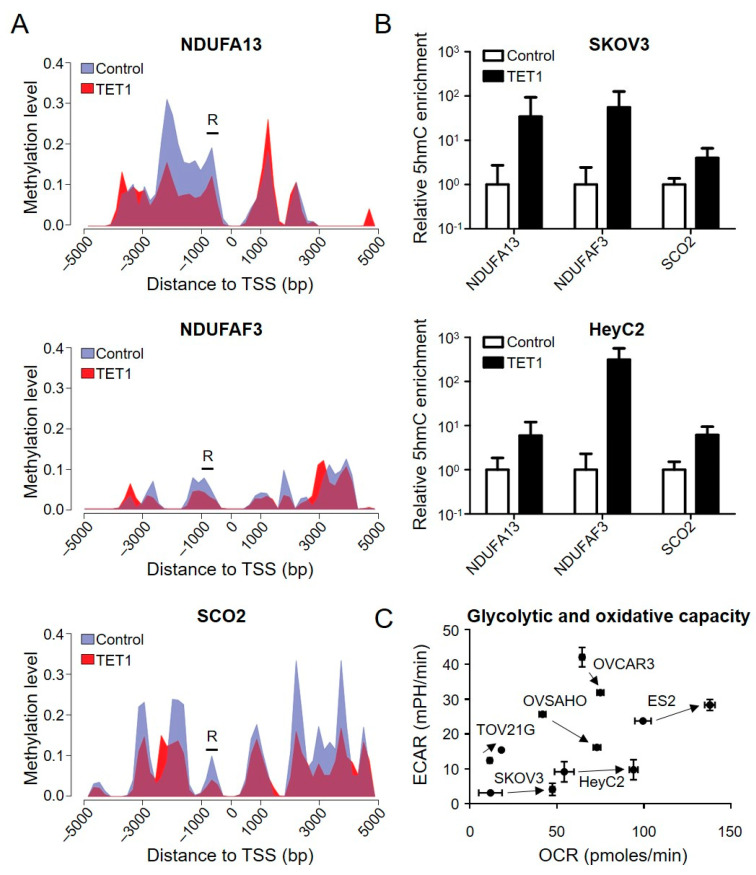
OXPHOS-related genes were demethylated through hydroxymethylation after TET1 expression. (**A**) Area charts display the methylation level of the control (hydrangea blue) and TET1-reprogrammed (red) HeyC2 cells. To visually represent the methylation changes, the stacked areas (non-overlapping areas) can be expressed as demethylation (hydrangea blue) or methylation (red) after TET1 expression. Black bars (R) show the 5gmC chromatin immunoprecipitation (ChIP) PCR regions, which contain a predicated TET1 binding site. (**B**) The 5-hmC levels of *NDUFA13*, *NDUFAF3*, and *SCO2* were measured using 5gmC ChIP PCR. The 5-hmC marker was enriched in the promoter regions of the target genes after TET1 expression. (**C**) TET1 increased the mitochondrial respiratory capacities of the ovarian cancer cells. The oxygen consumption rate (OCR) and extracellular acidification rate (ECAR) of TET1-reprogrammed SKOV3, HeyC2, ES2, TOV21G, OVCAR3, and OVSAHO were measured using the real-time Seahorse XF Extracellular Flux Analyser. TET1-reprogrammed CSCs had higher mitochondrial respiratory capacities than the control cells.

To examine whether TET1 could activate the OXPHOS-related genes using DNA demethylation, we assessed the methylation levels of the target genes from 5 kb upstream to 5 kb downstream of the transcription start site using MethylCap-seq. TET1 caused a dramatic decrease in the DNA methylation levels around the promoter regions of *NDUFA13*, *NDUFAF3*, and *SCO2*, with a reduction of 0.48-, 0.44, and 0.25-fold, respectively, as shown in the methylation area chart (Figure 2A and Supplementary Figure S2). To further confirm whether DNA demethylation occurred through a hydroxymethylation manner, we used β-glucosyl-5-hmC chromatin immunoprecipitation (5gmC ChIP)-qPCR to measure the 5-hmC levels of these genes’ demethylation region (R), where there is a predicted TET1 binding site. We found that TET1 enriched the 5-hmC levels in the *NDUFA13*, *NDUFAF3*, and *SCO2* promoter regions in SKOV3 and HeyC2 CSCs (Figure 2B). These findings indicate that TET1 directly reactivated the OXPHOS-related genes through hydroxymethylation-dependent DNA demethylation in ovarian cancer, implying that TET1 may reprogram the energy metabolism process in ovarian cancer, in which cells tend to use mitochondrial respiration to produce energy.

### 3.2. TET1 Enhanced Bioenergetic Properties and Mitochondrial Function of TET1-Reprogrammed Ovarian CSCs

To characterize the bioenergetics profile of TET1-reprogrammed ovarian CSCs (SKOV3, HeyC2, OVCAR3, OVSAHO, TOV21G, and ES2), we used the Agilent Seahorse XF Analyzer to determine the oxygen consumption rate (OCR) and extracellular acidification rate (ECAR), which are indicative of the mitochondrial OXPHOS and glycolytic rates, respectively. TET1 increased the cellular OCR (X-axis) in all TET1-reprogrammed ovarian CSCs, ranging from 1.16- to 4.00-fold (Figure 2C), indicating that TET1 switched the cellular energy metabolism to higher levels of OXPHOS, which is a more efficient state. Indeed, TET1-reprogrammed ovarian CSCs were more sensitive to the OXPHOS inhibitor, oligomycin, rather than the glycolysis inhibitor, 2-deoxy-D-glucose (Supplementary Figure S3). ECAR was reduced in high-grade serous carcinoma (OVCAR3 and OVSAHO), which indicated a dependence on mitochondrial respiration rather than glycolysis. ECAR and OCR were increased in clear cell carcinoma (ES2 and TOV21G), which suggested an equal use of both glycolysis and OXPHOS; however, ECAR was not affected in SKOV3 and HeyC2, which implied a preference for OXPHOS.

**Figure 3:**
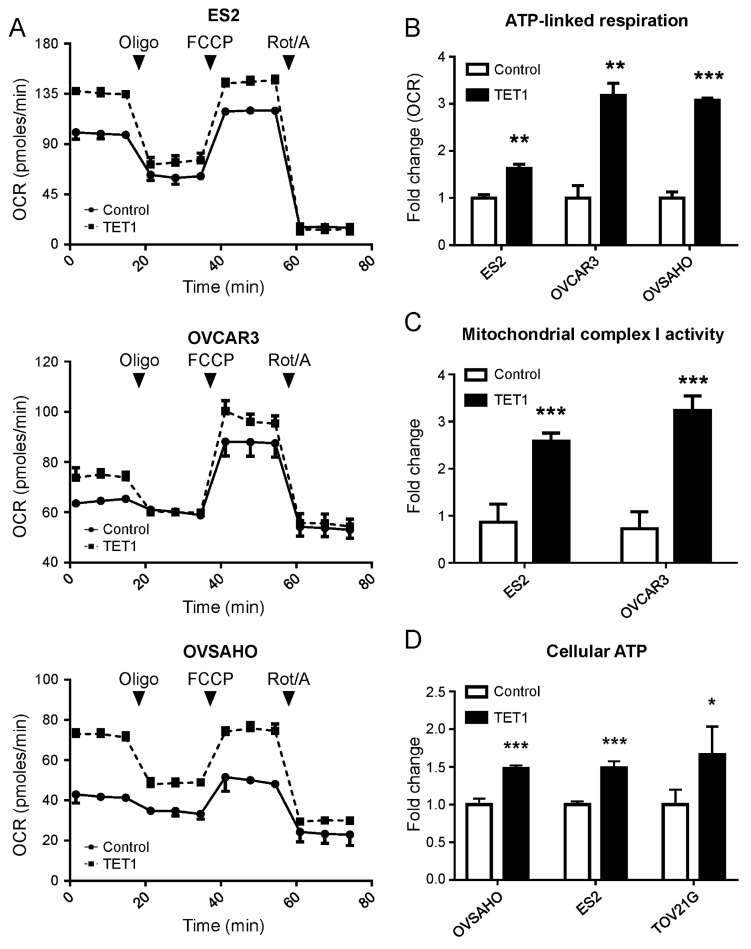
TET1 enhanced the bioenergetic properties and function of mitochondria. (**A**) The real-time mitochondrial respiratory profile was measured using the oxygen consumption rate (OCR) using the Seahorse XF Extracellular Flux Analyser. (**B**) The ATP-linked respiration was calculated, which was derived from the difference between the OCR at baseline and respiration of (**A**) following oligomycin addition. (**C**) The mitochondrial complex I activity of ovarian cancer cells was measured using the Mitochondrial Complex I Activity Assay Kit. TET1-reprogrammed CSCs displayed significantly higher complex 1 activity than the control cells. (**D**) Cellular ATP levels in ovarian cancer cells were measured using the ATP Determination Kit. TET1 increased cellular ATP levels in ovarian cancer. *, *p* < 0.05; **, *p* < 0.01; ***, *p* < 0.001.

To implicate TET1 expression more thoroughly in the alteration of mitochondrial energy production of TET1-reprogrammed ovarian CSCs, we evaluated the mitochondrial respiratory profiles using OCR measurements over time. The baseline OCR measurements were followed by the addition of oligomycin (injection 1) that inhibited complex V (mitochondrial ATP synthase), causing a decrease in the OCR. Then, carbonyl cyanide-p-trifluoromethoxyphenylhydrazone (injection 2) was added, which caused the uncoupling of mitochondrial OXPHOS and resulted in maximal respiration. Finally, a combination of rotenone and antimycin A (injection 3) was added to suppress the mitochondrial complex I and III activities of the mitochondrial respiration chain (Figure 3A). We found that TET1 increased basal and ATP-linked respiration capacities in ES2, OVCAR3, and OVSAHO CSCs, as well as maximal respiration capacities in ES2 and OVSAHO CSCs (Figure 3B and Supplementary Figure S4). Furthermore, we found that TET1 increased the mitochondrial complex I activity (Figure 3C) and cellular ATP levels (Figure 3D) in ovarian CSCs by 2.41- to 3.44-fold and 1.32- to 2.05-fold, respectively, using the Mitochondrial Complex I Activity Assay Kit and the ATP Determination Kit. Thus, TET1 enhanced mitochondrial complex I activity and OXPHOS capability to produce ATP in ovarian CSCs.

### 3.3. TET1 Enhanced Mitochondrial Biogenesis and Detoxification Capacity in TET1-Reprogrammed Ovarian CSCs

**Figure 4:**
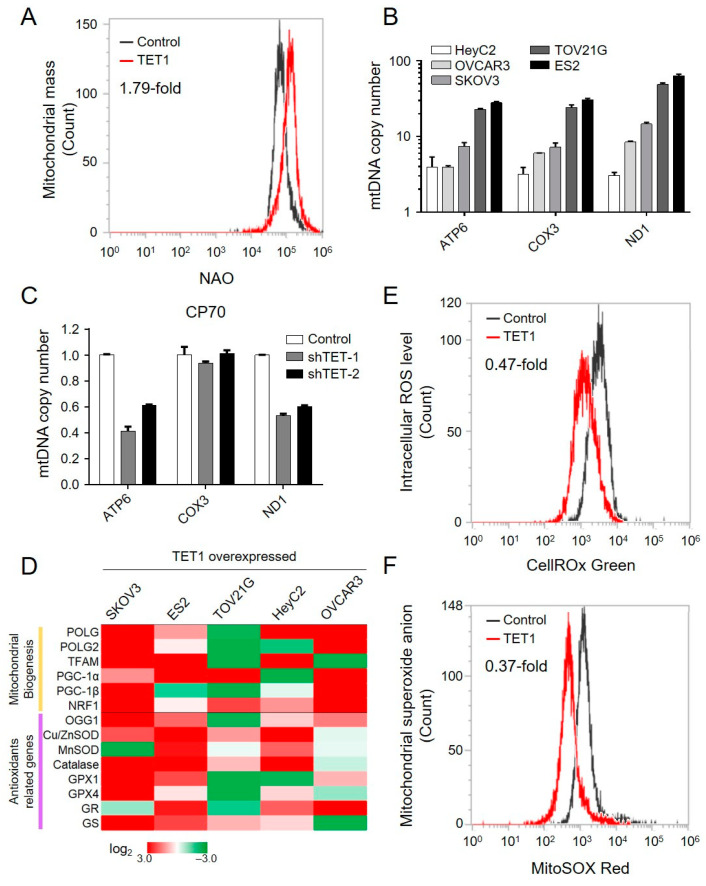
TET1 increased the mitochondrial biogenesis and detoxification capability. (**A**) The mitochondrial mass was assessed using the nonyl acridine orange (NAO) assay in ES2. (**B**) The mitochondrial copy number was measured using qPCR of the mitochondrial DNA, *ATP6*, *COX3*, and *ND1* in (**B**) TET1-reprogrammed and (**C**) TET1-knockdown cells. (**D**) The heat map shows the expression level of mitochondrial biogenesis and antioxidant-related genes using qPCR. (**E**) The intracellular reactive oxygen species and (**F**) superoxide anion levels in ES2 were measured using CellROX and MitoSOX staining, respectively.

The aforementioned findings supported a unique role for the elevated OCR levels of TET1-reprogrammed ovarian CSCs. Therefore, to determine whether TET1 increased the mitochondrial respiratory capacity by activating the OXPHOS pathway alone or in conjunction with an increased mitochondrial number, we investigated the mitochondrial mass using the 10-N-nonyl acridine orange assay and the mitochondrial DNA copy number (mtDNA-CN) using qPCR for mtDNA, ATP6, COX3, and ND1. We found that TET1 increased the mitochondrial mass by 1.79-fold in ES2 (Figure 4A). We also observed an increase in mtDNA-CN for all the different histotypes of ovarian cancer (Figure 4B). Consistently, by inhibiting TET1 expression in CP70, mtDNA-CN was decreased (Figure 4C). Gene expression profiles after TET1 expression in the different histotypes of ovarian cancer were further determined using qPCR. As expected, TET1 activated several mitochondrial biogenesis genes (Figure 4D).

The mitochondrial OXPHOS pathway is known to produce reactive oxygen species (ROS), such as superoxide anion (O_2_^−^). We next evaluated the intracellular ROS and O_2_^−^ levels using the CellROX Green and MitoSOX Red assays, respectively (Figure 4E,F). Interestingly, the intracellular levels of ROS and O_2_^−^ decreased by 0.47- and 0.37-fold, respectively, together with the upregulation of several antioxidant-related genes after TET1 expression (Figure 4D). Our findings indicated that TET1 enhanced mitochondrial biogenesis, increased mitochondrial mass, activated the OXPHOS pathway, and enhanced the antioxidant capability to reduce reactive oxygen species.

### 3.4. TET1-Reprogrammed Ovarian CSCs Increased Metabolites of the TCA Cycle, Especially α-KG

The TCA cycle intermediates, α-KG, succinate, and fumarate, can regulate the levels of DNA and histone methylation. DNA demethylases (TETs) are members of the α-KG-dependent hydroxylase family. Therefore, it is not surprising that TCA cycle metabolites can regulate gene expression by modifying epigenetic reprogramming.

**Figure 5:**
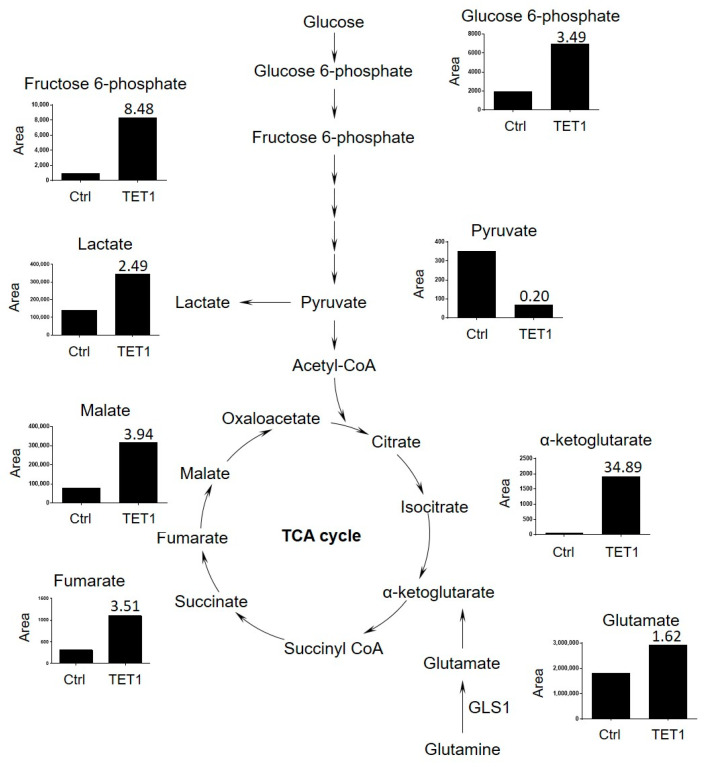
The tricarboxylic acid (TCA) cycle metabolites were significantly increased among TET1-reprogrammed ovarian CSCs. The TCA cycle metabolite levels (glucose-6-phosphate, fructose-6-phosphate, pyruvate, lactate, α-ketoglutarate, glutamate, fumarate, and malate) of control and TET1-reprogrammed ovarian CSCs are shown. The representative metabolite peak chromatograms are shown in Supplementary Figure S5.

We next applied mass spectrometry to detect the TCA cycle metabolites, including glucose-6-phosphate, fructose-6-phosphate, pyruvate, lactate, α-KG, glutamate, fumarate, and malate in the control and TET1- reprogrammed ES2 CSCs (Supplementary Figure S5). The TCA metabolites were significantly increased in TET1-reprogrammed ES2 CSCs, especially α-KG, which showed a 34.89-fold increase (Figure 5). We also found a marginal increase in glutamine uptake, by a 1.62-fold change. Our findings indicated that TET1 most significantly increased the levels of α-KG, which could maintain TET1 activity and, in turn, provide a positive regulatory loop for reprogramming metabolic epigenomes.

### 3.5. Inhibition of OXPHOS Affected Ovarian CSCs Growth and Had Synergistic Effects with CK2 Subunit Alpha (CK2α) Inhibitor

**Figure 6:**
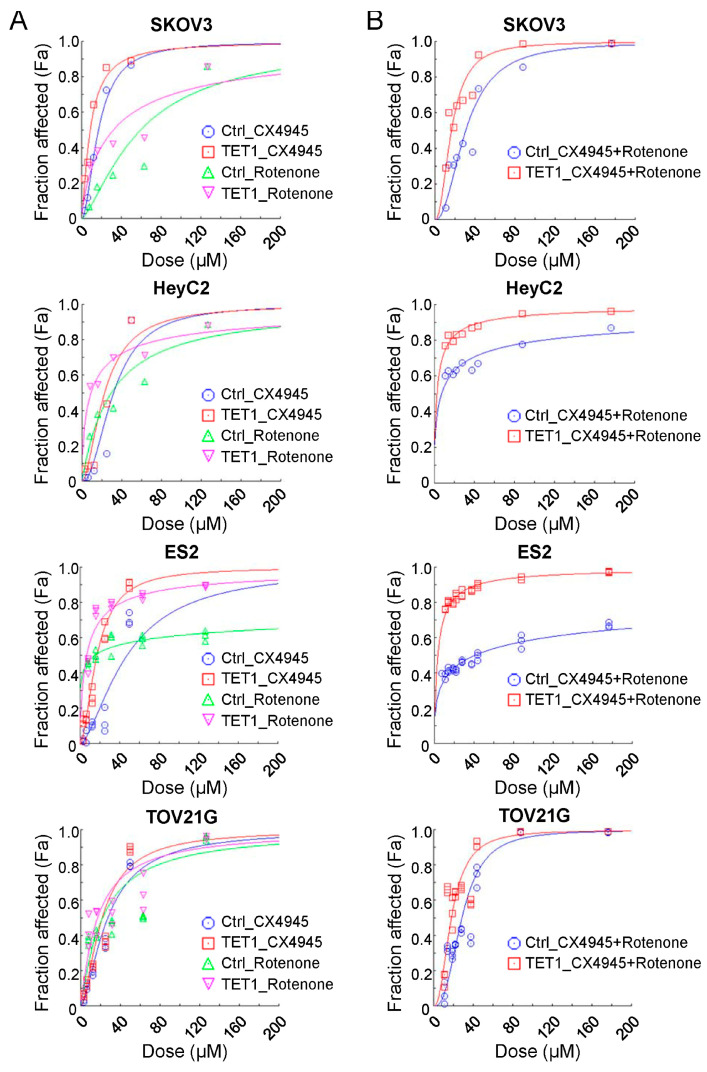
Combination effect of therapies targeting the mitochondria and casein kinase 2 pathways. Ovarian cancer cell dose–effect curves simulated by the median-effect equation for CX-4945 and rotenone using CompuSyn software with (**A**) actual single and (**B**) combination drug data points are shown. The left-shift curves indicate that the cells were susceptible to the drug treatment.

Considering that TET1-reprogrammed ovarian CSCs showed increased mitochondrial respiration and enhanced complex I activity, we examined the growth inhibition of the mitochondrial complex I inhibitor, rotenone, in TET1-reprogrammed ovarian CSCs. We examined the dose–response curves and half-maximal inhibitory concentration values of rotenone (Figure 6A, green and pink curves) in four ovarian cancer cells. The half-maximal inhibitory concentration values (μM) of rotenone were reduced by 0.54-, 0.30-, 0.45-, and 0.77-fold in TET1-reprogrammed SKOV3, HeyC2, ES2, and TOV21G CSCs, respectively (Supplementary Table S1). Our previously published data strongly suggested that TET1-reprogrammed ovarian CSCs were also susceptible to the CK2α inhibitor, CX-4945 (Figure 6A, blue and red curves). Therefore, we hypothesized that simultaneous targeting by the mitochondrial complex I inhibitor and the TET1-mediated immune modulator CK2α may have additive or synergistic effects on ovarian cancer cell growth inhibition. Next, we calculated the combination index and fraction-affected values of rotenone and CX-4945 by integrating the survival data using the CompuSyn algorithm (Supplementary Table S2). Highly synergistic and dose-dependent treatment effects of a combination of rotenone and CX-4945 were observed in different TET1-reprogrammed ovarian CSCs (Figure 6B). The combination of CX-4945 and rotenone translated into a 13.68- and 6.28-fold reduction in the dose required to achieve 75% cell death for CX-4945 and rotenone, respectively, as compared with single-agent treatment in TET1-reprogrammed HeyC2 CSCs (Supplementary Figure S6 and Table S3). Thus, our data indicated that the combined treatment of rotenone and CX-4945 in TET1-reprogrammed ovarian CSCs resulted in significant synergistic effects as compared with control cells under different drug concentration conditions.

## 4. Discussion

The metabolism of CSCs remains rarely investigated, especially in ovarian cancer. Cancer cells engage metabolic pathways, commonly known as the ‘Warburg effect’ [24,25]; however, the Warburg effect is not a feature of all types of cancers, or even of all cancer cells in the same tumor, because cancer cells are heterogeneous [22,23]. Importantly, altered metabolism and epigenome interactors in malignancies are likely to contribute to the generation of CSCs [30]. Some studies have suggested that CSCs display decreased mitochondrial function and an increased dependence on glycolysis [35], while others reported an increased dependence on mitochondrial function and OXPHOS [36]. Lipid metabolism and glutaminolysis also play important roles in CSC maintenance [29,31]. Under normoxic conditions, CSCs generate most of the ATP through OXPHOS and the β-oxidation of fatty acids; however, under hypoxic conditions, the glycolytic rate in CSCs tends to be upregulated to generate energy and promote stemness properties. The metabolism of ovarian CSCs has been controversial [37,38,39]. Park et al. suggested that an inhibitor of pyruvate kinase M2 suppressed SKOV3 cell growth by targeting the Warburg effect [40]. Other studies have demonstrated that ovarian cancer cell lines use OXPHOS as a highly metabolic phenotype [38,41]. Moreover, glutamine [42,43] and fatty acid metabolism [44] were also reported as alternative energy sources for ovarian CSCs; however, the ovarian cancer stem status and the energy metabolic shift between OXPHOS, glycolysis, glutaminolysis, or fatty acid metabolism in ovarian CSCs remain unclear. Our findings demonstrated that, epigenetically, TET1 reprogrammed ovarian cancer and altered the mitochondrial OXPHOS pathway to activate respiration rather than glycolysis for energy production, suggesting metabolic plasticity via epigenetic regulation in ovarian CSCs.

Indeed, the epigenetic effects on metabolism and vice versa remain an intriguing issue. *TET2* mutations drive tumorigenesis in several cancers, particularly in myeloid malignancies [45,46]. Acute myeloid leukemia with mutations in *TET2* or *IDH2* is sensitive to epigenetic therapy involving the inhibition of DNA methyltransferase activity using 5-azacytidine or the inhibition of mutant *IDH2*. These inhibition methods induce a differentiation response and serve as a targetable mechanism-based combination therapy [44]. Metabolic heterogeneity and epigenetics were not well recognized until recently. Certain metabolic alterations occur at the epigenetic level. Moreover, for the metabolites that are generated during the glycolytic cycle, TCA and OXPHOS serve as cofactors that catalyze epigenetic modifications and transcriptional regulation [32]. Most demethylation reactions are catalyzed by a large family of α-KG-dependent dioxygenases, which includes the TET enzymes [47,48,49]. TET enzymatic activity depends on the availability of the co-substrates (α-KG, ferrous iron, and oxygen) and the presence of competitive inhibitors, such as 2-hydroxyglutarate, succinate, and fumarate [50]. The present study revealed that TET1-reprogrammed ovarian CSCs increased the metabolite levels of α-KG, which may reciprocally regulate the TET1 dioxygenase function.

Mitochondrial OXPHOS produces ATP through the electron transport complexes I, II, III, IV, and V [51]. The inhibition of these complexes is an effective strategy for the inhibition of OXPHOS and has been proposed as a potential therapeutic approach [37]. As the first step of the electron transport chain, the proton gradient is generated via the proton pump function of mainly complex I [52,53]. In new drug development, complex I is thus a target for inhibiting OXPHOS. A clinical-grade small molecule inhibitor of complex I, IACS-010759, was effective for tumor growth inhibition in mantle cell lymphoma [54]. Recently, a phase I trial of IACS-010759 in patients with advanced solid tumors, relapsed/refractory acute myeloid leukemias, and lymphomas is ongoing [55,56,57]. Accordingly, OXPHOS has emerged as a target for treating OXPHOS-dependent malignancies, such as leukemias, lymphomas, pancreatic ductal adenocarcinoma, specific subtypes of melanoma, and endometrial carcinoma [55]. Moreover, complex I inhibitors have been proposed to alter the microenvironment immune status [57,58], which may have implications in immunotherapy; however, novel applications of complex I inhibitors with a suitable therapeutic index to target ovarian cancer cell metabolism remain lacking [59]. Our findings supported the potential use of complex I inhibitors in future ovarian cancer treatment, especially in the TET1-high expression subgroup. In conjunction with our present and previous studies [9], we further explored the synergistic effects of the mitochondrial complex I inhibitor (rotenone) and the CK2α inhibitor (CX-4945) in different ovarian cancer cell lines. Although, in the present study, we did not evaluate the interactions between mitochondrial DNA and TET1, nor did we explore the role of TET1 in the regulation of mitochondrial morphology and dynamics; understanding how TET1 influences mitochondrial epigenetics and dynamics could provide a more comprehensive understanding of ovarian cancer metabolism and remains to be determined in future studies. Our findings provide evidence for combining these two novel strategies, metabolic intervention, and immune modulation, for ovarian cancer treatments in the future.

## 5. Conclusions

The present study revealed that TET1 could demethylate the OXPHOS genes, increase mitochondrial numbers, and shift the energy source to OXPHOS rather than glycolysis, which is a reverse phenomenon of the Warburg effect. We also demonstrated growth inhibition using a complex I inhibitor. Our findings contribute to the understanding of metabolic plasticity in TET1-reprogrammed ovarian CSCs and suggest a new therapeutic strategy involving metabolic intervention. Translational research for the clinical application and an understanding of the feedback loop between metabolic and epigenetic machinery in ovarian cancer are warranted.

## Data Availability

The RNA-seq data have been deposited in the GEO database under the accession code: GSE128098.

## Supplementary Materials

The following supporting information can be downloaded at: https://www.mdpi.com/article/10.3390/antiox13060735/s1, Figure S1: Relative TET1 mRNA expression levels in EOC cell lines; Figure S2: OXPHOS-related genes were demethylated after TET1 expression; Figure S3: TET1-reprogrammed ovarian CSCs tended to use mitochondrial respiration as their energy source; Figure S4: The Seahorse assay demonstrated the differences in mitochondrial function between control and TET1-reprogrammed ovarian CSCs; Figure S5: The TCA cycle metabolites were detected using mass spectrometry; Figure S6: Combination index (CI) analysis of ovarian CSCs after TET1 expression; Table S1: IC_50_ values of Rotenone treatment; Table S2: CI values of combination treatment; Table S3. IC_75_ value of single and combination treatment and drug-dose reduction.

## Abbreviations

5-hmC: 5-hydroxymethylcytosine; 5-mC: 5-methylcytosine; ATP: adenosine triphosphate; α-KG: alpha ketoglutarate; ChIP: chromatin immunoprecipitation; CI: combination index; CK2α: casein kinase-2 subunit alpha; CSC: cancer stem cell; DRI: dose-reduction index; ECAR: extracellular acidification rate; Fa: fraction affected; mtDNA: mitochondrial DNA; mtDNA-CN: mitochondrial DNA copy number; O_2_^-^: superoxide anion; OCR: oxygen consumption rate; OXPHOS: oxidative phosphorylation; ROS: reactive oxygen species; TCA cycle: tricarboxylic acid cycle; TCGA: The Cancer Genome Atlas; TET: ten-eleven translocation; TSS: transcription start site.
