# Supplementary material for: Active DNA Demethylase, TET1, Increases Oxidative Phosphorylation and Sensitizes Ovarian Cancer Stem Cells to Mitochondrial Complex I Inhibitor
Source: Antioxidants (Basel). 2024 Jun 17;13(6):735. doi: 10.3390/antiox13060735 (PMC11200674; doi:10.3390/antiox13060735)
Supplement: Supplementary file 1 [file antioxidants-13-00735-s001.zip › antioxidants-3001248-supplementary.pdf]

Supplementary figures legends

Figure S1. Relative TET1 mRNA expression levels in EOC cell lines. TET1 expression levels were normalized to TBP and compared via fold-changes from normal fallopian tubes (FE25, set as 1) in EOCs, as assessed by real-time PCR. Each bar represents the average of triplicate reactions ± SD.

Figure S2. OXPHOS-related genes were demethylated after TET1 expression. Area charts display the methylation level of control (hydrangea blue) and TET1-reprogrammed (red) cells. For visually representing methylation changes, the stacked areas (non-overlapping areas) can be expressed as demethylation (hydrangea blue) or methylation (red) after TET1 expression. Black bars show the demethylation regions.

Figure S3. TET1-reprogrammed ovarian CSCs tended to use mitochondrial respiration as their energy source. Control and TET1-reprogrammed ovarian cancer CSCs were treated with an OXPHOS inhibitor, oligomycin, as well as a glycolysis inhibitor, 2-deoxy-D-glucose (2-DG). TET1-reprogrammed ovarian CSCs showed more sensitivity to oligomycin rather than 2-DG. The cell viability was measured and presented using mean values ± SD of triplicate experiments. Control was set as 1 (100%). The *p*-values were measured using Student's t-test.

Figure S4. The Seahorse assay demonstrated the differences in mitochondrial function between control and TET1-reprogrammed ovarian CSCs. The basal respiration was derived by subtracting non-mitochondrial respiration. Next, oligomycin, which is a complex V inhibitor, was added and the resulting OCR was used to derive the ATP-linked respiration by subtracting the oligomycin OCR from baseline cellular OCR. The proton leak respiration was assessed by subtracting non-mitochondrial

respiration from the oligomycin rate. Next, carbonyl cyanide-p-trifluoromethoxyphenylhydrazone (FCCP), which is a protonophore, was added to collapse the inner membrane gradient, thereby allowing the electron transport chain to function at its maximal rate, and the maximal respiratory capacity was derived by subtracting the non-mitochondrial respiration from the FCCP OCR. Finally, rotenone and antimycin A, which are inhibitors of complexes I and III, were added to shut down the electron transport chain function, revealing the non-mitochondrial respiration. Mitochondrial reserve capacity was calculated by subtracting basal respiration from the maximal respiratory capacity.

Figure S5. The TCA cycle metabolites were detected using mass spectrometry. The representative peak chromatograms of glutamate, lactate, malate, pyruvate, glucose-6-phosphate, fumarate and α-ketoglutarate are shown.

Figure S6. Combination index (CI) analysis of ovarian CSCs after TET1 expression. Fraction affected (Fa) versus CI plots were generated using the Chou–Talalay method to determine the extent of synergy for a combination of CX-4945 and rotenone in ovarian cancer cell lines. Synergistic effects are defined as CI < 1, additive effects as CI = 1 and antagonistic effects as CI > 1. The dotted line in each plot indicates a reference point of a CI value of 1. Fa = 0.5 indicates 50% cell death and Fa = 0.8 indicates 80% cell death.

Supplementary Figure S1

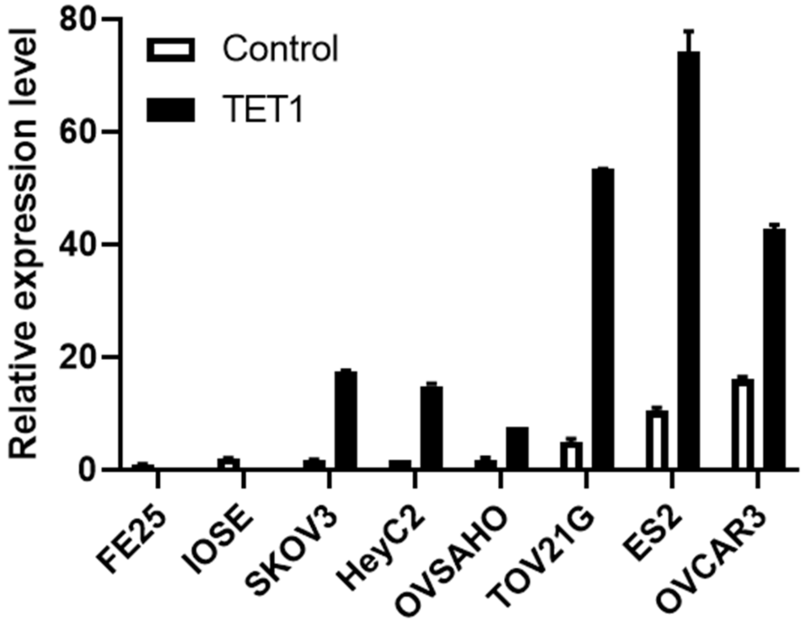

Supplementary Figure S2

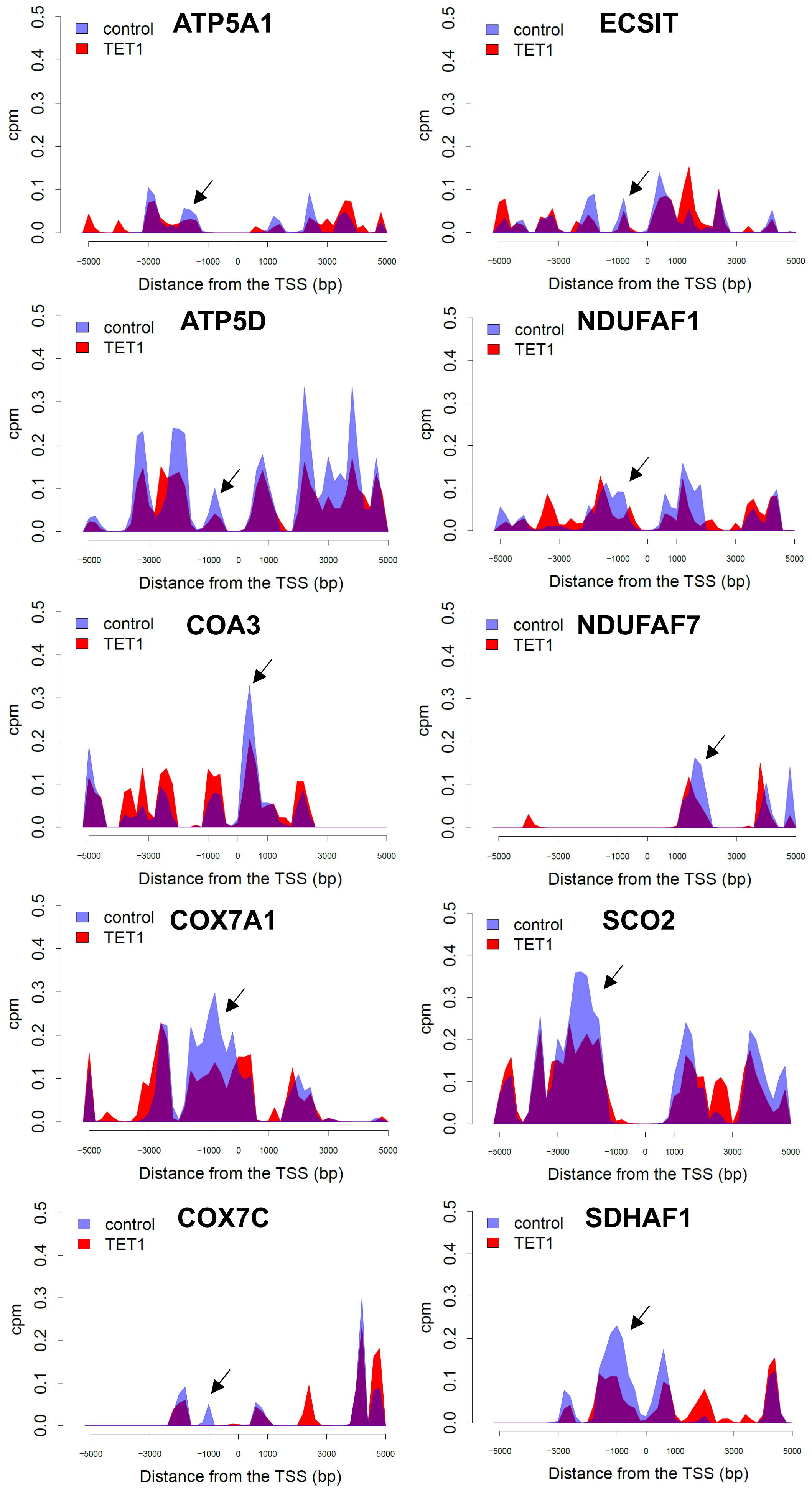

Supplementary Figure S3

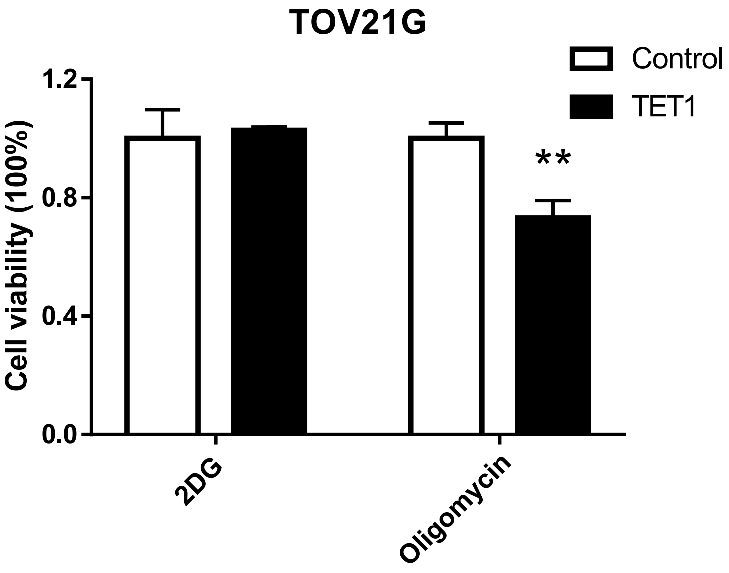

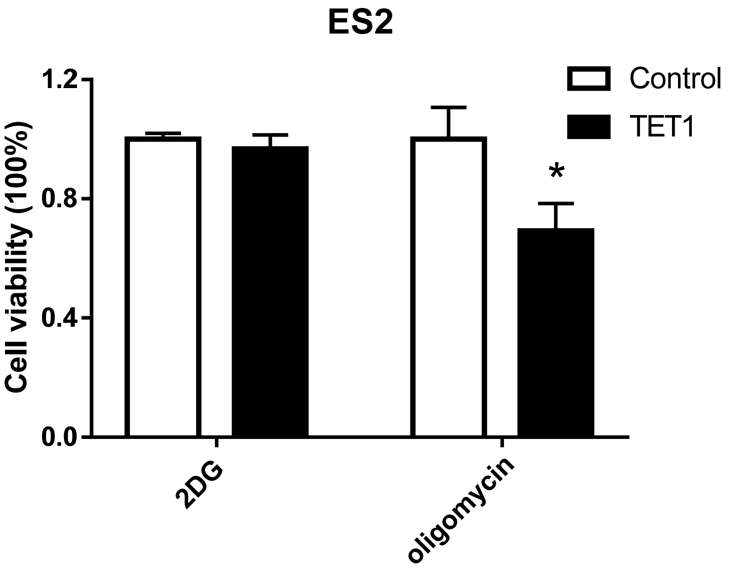

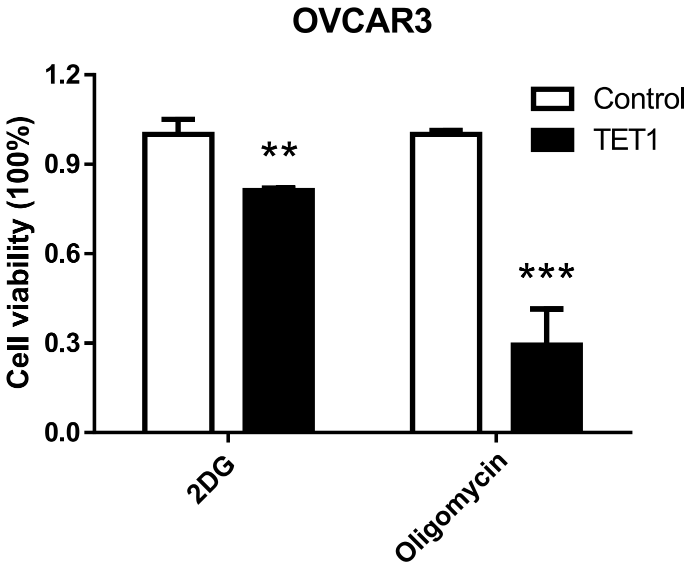

Supplementary Figure S4

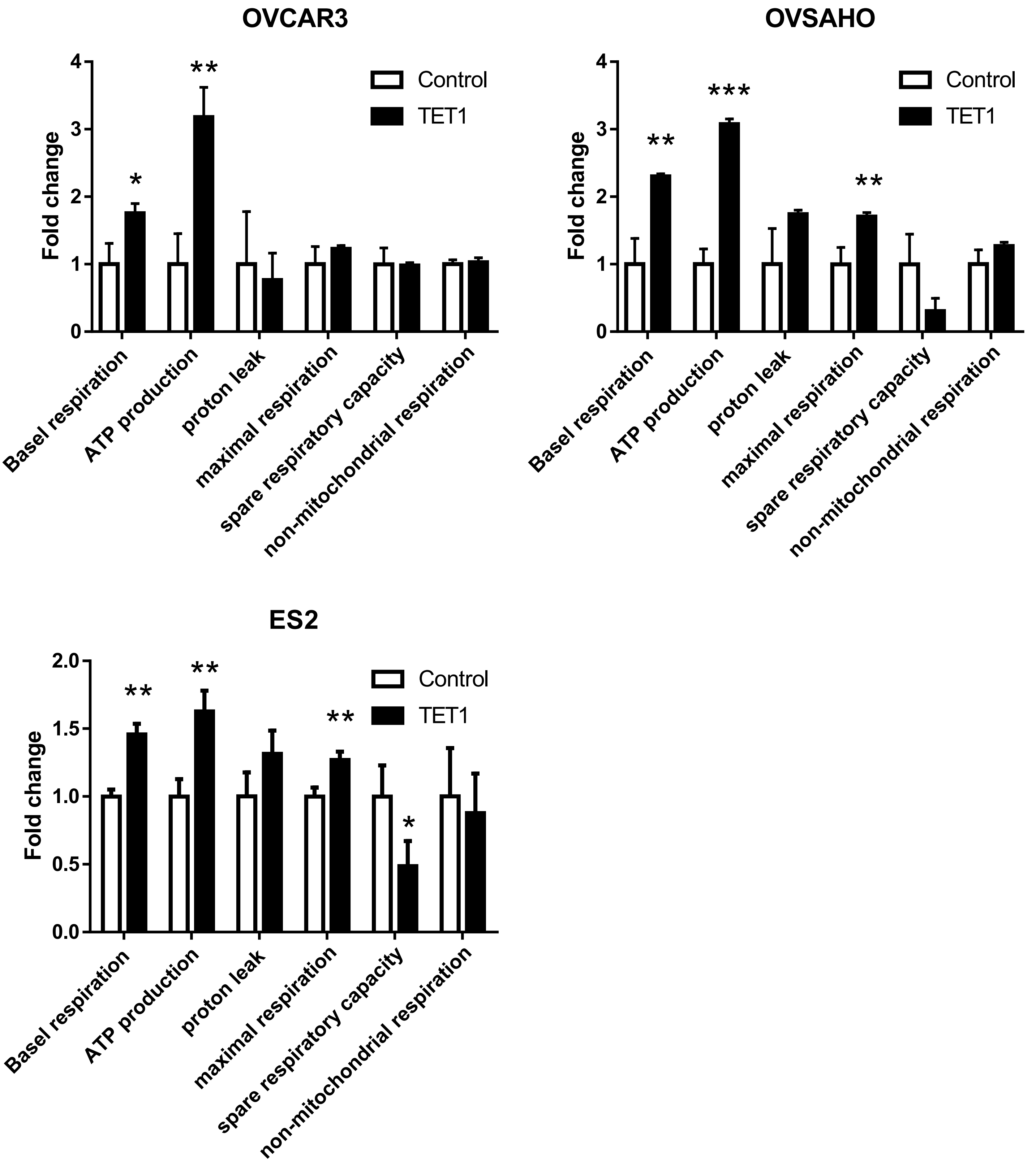

Supplementary Figure S5

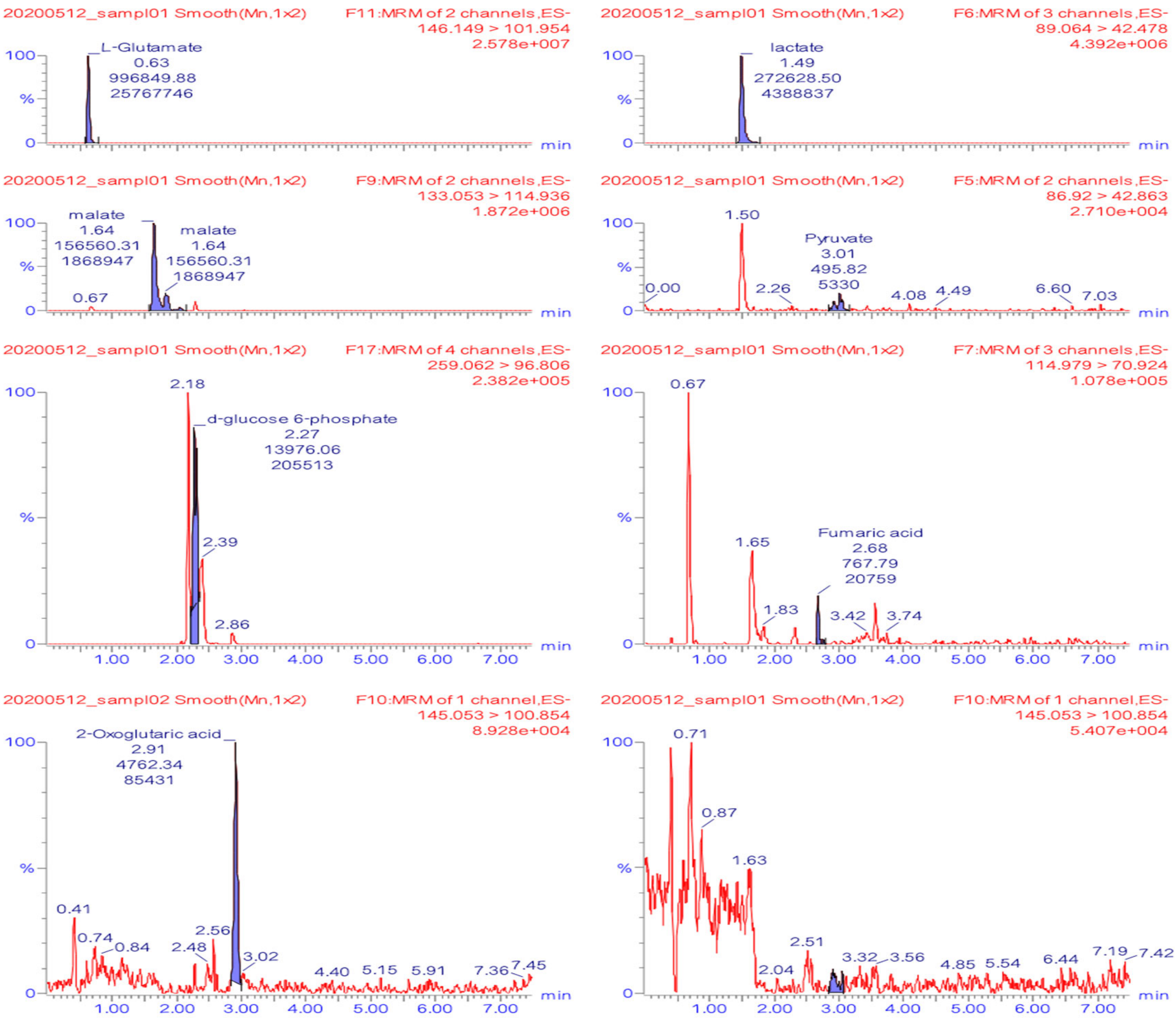

Supplementary Figure S6

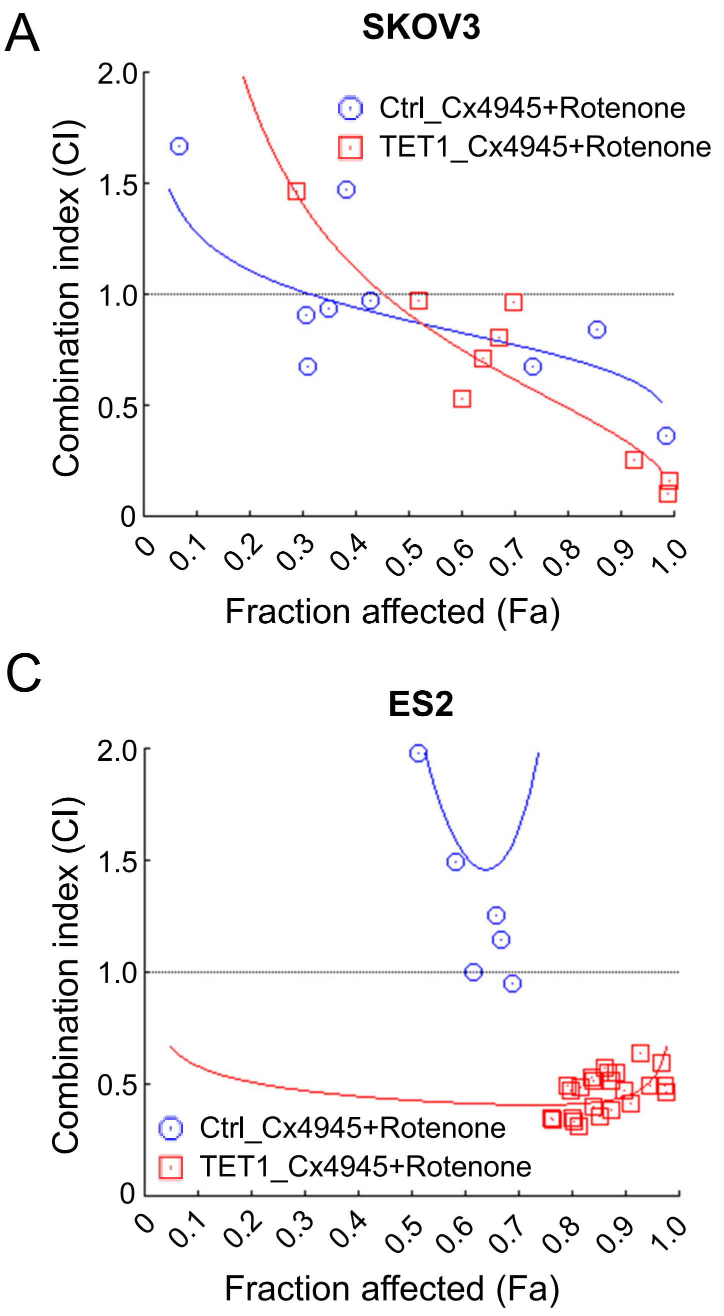

Table S1. IC_{50} values of Rotenone treatment (μM)

	Control	TET1	Fold change
SKOV3	59.174	31.650	0.535
HeyC2	29.197	8.860	0.303
ES2	14.565	6.534	0.449
TOV21G	21.734	16.758	0.771

Table S2. CI values of combination treatment (μM)

Fa	SKOV3		HeyC2		ES2		TOV21G	
	Control	TET1	Control	TET1	Control	TET1	Control	TET1
0.5	0.884	0.913	0.239	0.250	2.238	0.430	1.235	1.000
0.6	0.829	0.753	0.374	0.238	1.522	0.418	1.023	0.849
0.7	0.775	0.617	0.616	0.232	1.647	0.411	0.836	0.712
0.8	0.715	0.490	1.150	0.236	3.371	0.414	0.656	0.578
0.9	0.636	0.355	3.047	0.277	14.362	0.449	0.459	0.425

Table S3. IC_{75} value of single and combination treatment and drug-dose reduction (μM)

		Control		TET1	
	Fa=0.75	CX4945	Rotenone	CX4945	Rotenone
SKOV3	Single	30.76	131.82	19.74	123.47
	Combined	14.42	36.55	7.77	19.69
	DRI	2.13	3.61	2.54	6.27
	CI	0.746		0.553	
HeyC2	Single	53.39	87.04	42.78	49.81
	Combined	17.18	43.56	3.13	7.93
	DRI	3.11	2.00	13.68	6.28
	CI	0.822		0.232	
ES2	Single	94.59	1428.06	31.82	28.50
	Combined	176.30	446.95	3.41	8.65
	DRI	0.54	3.20	9.33	3.29
	CI	2.177		0.411	
TOV21G	Single	55.17	59.36	43.08	45.93
	Combined	12.26	31.08	8.24	20.872
	DRI	4.50	1.91	5.23	2.20
	CI	0.746		0.646	

DRI: dose-reduction index
